# Mechanical strain induces involution-associated events in mammary epithelial cells

**DOI:** 10.1186/1471-2121-10-55

**Published:** 2009-07-17

**Authors:** Ana Quaglino, Marcelo Salierno, Jesica Pellegrotti, Natalia Rubinstein, Edith C Kordon

**Affiliations:** 1 Departamento de Química Biológica e Instituto de Fisiología, Biología Molecular y Neurociencias (IFIBYNE)-CONICET, Facultad de Ciencias Exactas y Naturales, Universidad de Buenos Aires, Argentina; 2 Departamento de Química Inorgánica, Analítica y Química Física, INQUIMAE-CONICET, Facultad de Ciencias Exactas y Naturales, Universidad de Buenos Aires, Buenos Aires, Argentina

## Abstract

**Background:**

Shortly after weaning, a complex multi-step process that leads to massive epithelial apoptosis is triggered by tissue local factors in the mouse mammary gland. Several reports have demonstrated the relevance of mechanical stress to induce adaptive responses in different cell types. Interestingly, these signaling pathways also participate in mammary gland involution. Then, it has been suggested that cell stretching caused by milk accumulation after weaning might be the first stimulus that initiates the complete remodeling of the mammary gland. However, no previous report has demonstrated the impact of mechanical stress on mammary cell physiology. To address this issue, we have designed a new practical device that allowed us to evaluate the effects of radial stretching on mammary epithelial cells in culture.

**Results:**

We have designed and built a new device to analyze the biological consequences of applying mechanical stress to cells cultured on flexible silicone membranes. Subsequently, a geometrical model that predicted the percentage of radial strain applied to the elastic substrate was developed. By microscopic image analysis, the adjustment of these calculations to the actual strain exerted on the attached cells was verified. The studies described herein were all performed in the HC11 non-tumorigenic mammary epithelial cell line, which was originated from a pregnant BALB/c mouse. In these cells, as previously observed in other tissue types, mechanical stress induced ERK1/2 phosphorylation and c-Fos mRNA and protein expression. In addition, we found that mammary cell stretching triggered involution associated cellular events as Leukemia Inhibitory Factor (LIF) expression induction, STAT3 activation and AKT phosphorylation inhibition.

**Conclusion:**

Here, we show for the first time, that mechanical strain is able to induce weaning-associated events in cultured mammary epithelial cells. These results were obtained using a new practical and affordable device specifically designed for such a purpose. We believe that our results indicate the relevance of mechanical stress among the early post-lactation events that lead to mammary gland involution.

## Background

Cells are able to act in response to multiple biochemical and biophysical stimuli, depending on their type and function. Particularly, it has been shown that mechanical forces trigger specific events in a variety of cell types under different physiological and pathological situations [1-5]. For example, it has been demonstrated that in solid tumors, cells are subjected to mechanical stress due to elevated interstitial pressure and perturbed vasculature [6]. The dramatic consequences of this strain were demonstrated when it was shown that extracellular matrix rigidity induced malignant phenotype in mammary epithelial cells [7].

In the mouse mammary gland, post-lactational involution is divided in two distinct phases. The first one starts only hours after weaning, when milk stasis induces the expression of local factors that trigger apoptosis of the alveolar epithelium [8,9]. Then, after a few days, there is a decline in the lactogenic hormone circulating levels and tissue remodeling of the mammary gland is initiated [10]. Interestingly, the specific signals that derive from milk stasis and result in the release of those mammary local factors remain unknown. It has been proposed that milk accumulation, caused by the lack of suckling, might subject alveolar cells to mechanical strain. This stress could be, *per se*, the earliest stimulus to trigger expression and release of local factors that would initiate mammary gland involution [11]. To address this question, we developed a new device, inspired in a system previously described by Lee and coworkers [12], which allowed us to exert up to 30% radial strain to non-tumorigenic mammary epithelial cells (HC11). Using this device we observed that radial cell stretching modified the expression and/or activation of c-Fos, ERK1/2, AKT, STAT3 and Leukemia Inhibitory Factor (LIF), which are involved in mammary regression after weaning. These results confirmed that mechanical stress could be the very first initiator of post-lactational mammary gland involution.

## Methods

### Equibiaxial stretching device

**Figure 1:**
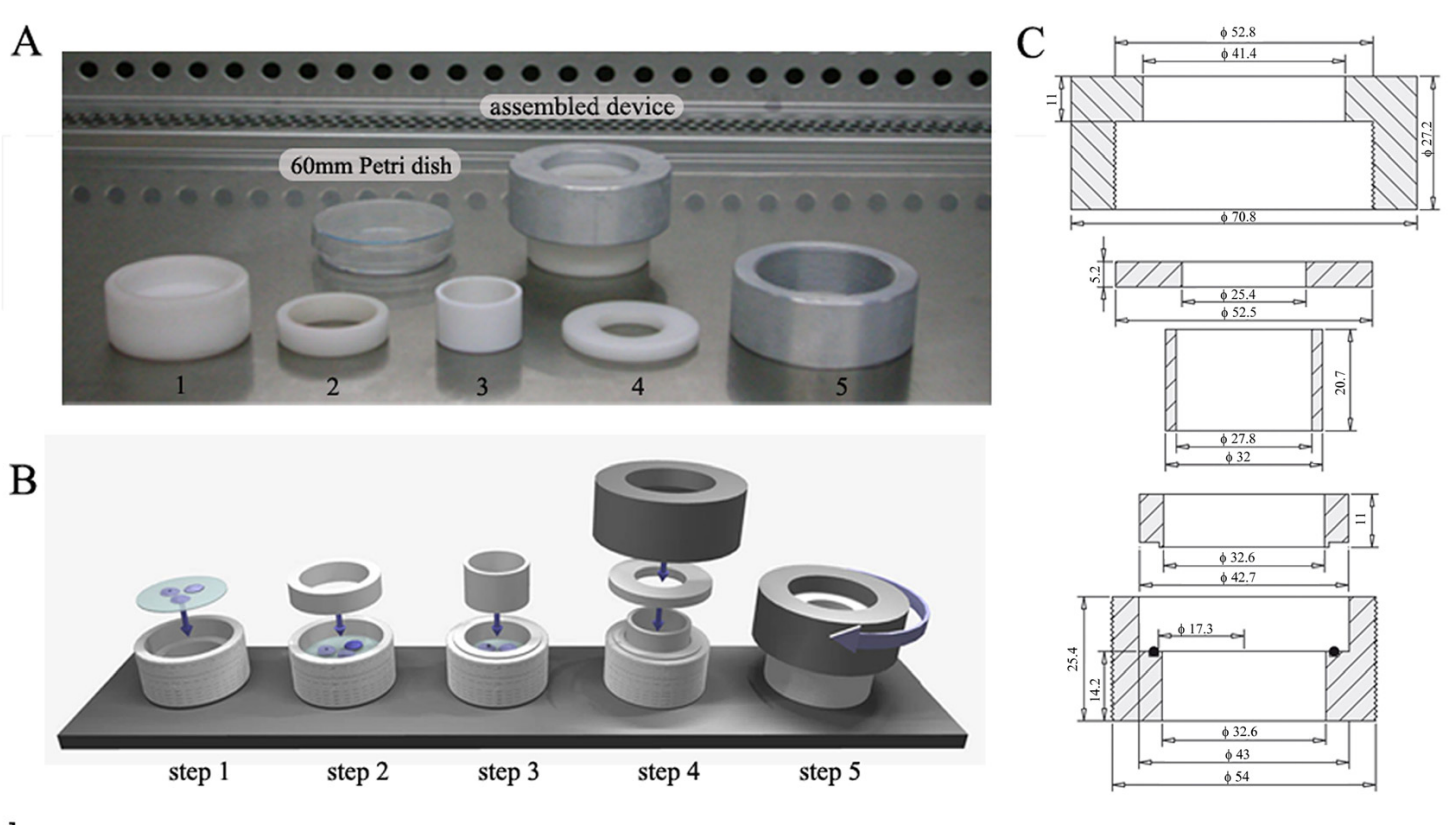
**Equibiaxial stretching device**. (A) Assembled device and its individual components (1–5): 1) Delrin^® ^made cylinder with an inner ring that constitutes the silicone membrane holder; 2) Delrin^® ^made ring with an O-ring that attaches the membrane to the inner holder; 3) Teflon^® ^made indenter ring; 4) Delrin^® ^made flange that pushes down piece #3; 5) Aluminum screw-top, that pushes down piece #4. (B) Diagram (in scale) depicting the procedure to assemble the complete device for performing cell stretching protocols; step 1 indicates positioning of the flexible membrane with attached cells grown in a 60 mm Petri dish; following steps indicate assembly of components shown in (A); (C) Cross-section of the complete device. For each piece, specific dimensions are described in millimeters.

The device designed to apply controlled equibiaxial strain to cells attached to stretchable membranes, consist in 5 different pieces (Figure 1A):

1) Delrin^® ^made cylinder (item #1) that constitutes the bottom of the assembled device. This piece has a protruding ring in the inner face, where the silicone membrane (43 mm diameter) is placed.

2) Delrin^® ^made ring (item #2) with an O-ring that fits the inner membrane holder. This piece is placed inside item #1 and attaches the membrane to the inner holder.

3) Indenter ring (item #3, Teflon^® ^made), which fits in item #2.

4) Flange (item #4, Delrin^® ^made) that pushes down the indenter ring (item #3).

5) Aluminum screw-top (item #5) that, when turned down, pushes the indenter ring (item #3) that stretches the silicone membrane.

The assembly process is described in Figure 1B. Complete specifications for this apparatus are detailed in Figure 1C. The 3D device model shown in Figure 1B was built using Rhinoceros software version 3.0 (McNeel North America, Seattle, USA). In addition, for practical purposes, we calculated the relationship between the indentor penetration and number of thread turns, which is 0.54 mm/turn.

Selection of materials for building this device and the stretchable membranes was made according to previous reported apparatus [12,13]. Since they resist high temperatures without loosing their physical properties, these devices can be sterilized by autoclaving.

It must be taken into consideration that in this device, cell medium is in close contact with the Teflon^® ^made indenter (item #3). Perfluorooctanoic acid (PFOA) is a chemical compound that was detected in trace amounts in finished Teflon products. The Agency's Science Advisory Board of the Environmental Protection Agency (EPA) suggested that PFOA is "likely to be carcinogenic to humans". However, EPA is still in the process of evaluating this information and has not made any definitive conclusions at this time . This should be considered for apparatus manipulation and if the device were used for evaluating effects that could be altered or imitated by traces of this compound.

### Laboratory-made silicone membranes

Elastic silicone membranes were made by vulcanizing liquid silicone rubber (RhodorsilRTV-1556, Rhodia, France) with Platinum as catalyst. Preformed matrices were filled with this material that polymerized at room temperature (25°C) during 3–7 days. Later, membranes were treated with 5.7% KOH in methanol for 5 min to neutralize the polymerization-derived HCl. After being washed with double-distilled water, silicone membranes were sterilized by autoclaving. Cell attachment was facilitated by incubating membranes with 50 μg/ml of rat collagen type I (Sigma, CA) in 0.02N acetic acid for 1 h at room temperature. Then, membranes were rinsed with phosphate-buffered saline solution and cells were seeded.

### Cell culture

The HC11 cell line, derived from pregnant BALB/c mouse mammary glands, was maintained as previously described [14]. To perform the experiments, comercially available silicone membranes (Flexcell International, Hillsborough, NC, USA) or laboratory-made silicone membranes, both coated with collagen type I, were placed inside Petri dishes (60 mm diameter). Cells were plated at a density of 7 × 10^5 ^cells/dish. After attachment, they were grown for approximately 48 h, until the cell culture became confluent. Then, culture medium was removed and cells were washed and held for 12 h under serum-free conditions.

### Mechanical Stimulation

Membranes with the attached cells were removed from the culture dishes and carefully placed inside the stretching device (Figure 1B, step 1). Then, the unit was assembled by the steps (1 to 5) described in Figure 1B and pre-warmed serum-free culture medium was added on top of the cells. Membranes were either exposed to different intensities (0 to 30%) of sustained radial strain for 1 h, or to 20% radial strain for different time intervals (15 min to 3 h). Afterwards, mRNA and protein extraction protocols were carried out as described below.

### Theoretical Model

**Figure 2:**
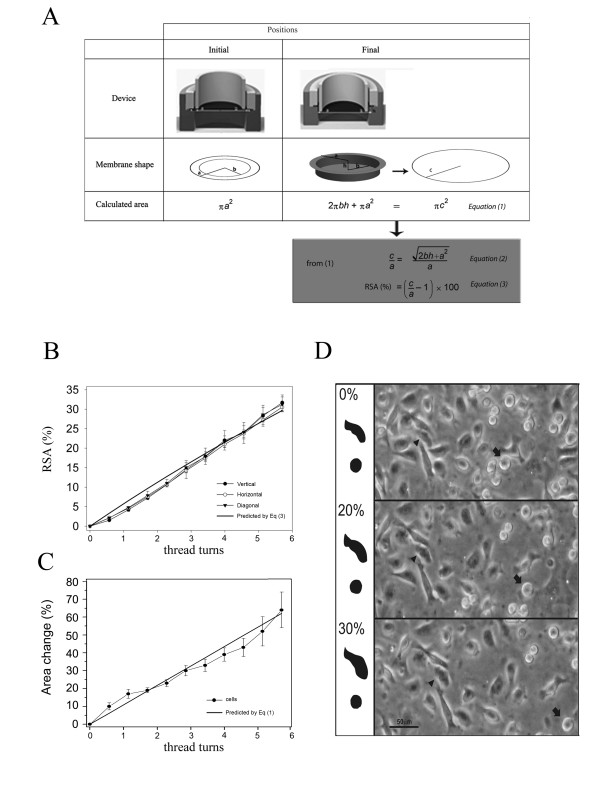
**Theoretical model to predict membrane strain magnitude**. (A) Initial and final position of stretching device (1st row) and flexible membrane (2nd row); equations describing membrane area in each position (3rd row); *a*: radius of circular stretchable area; *b*: radius of inner circle where cells can be visualized; *h*: distance made by the flange (item#4 of Figure 1); *c*: radius of a hypothetical new circle corresponding to the area of the stretched membrane in the final position (Equation (1)). The relative increment of *c/a *can be calculated by *Equation (2)*. *Equation (3) *indicates the percentage of radial strain applied (RSA) to the membrane. (B) The distances between 10 pairs of cells were measured in each orientation (horizontal, vertical and diagonal) in 4 different microscope fields. Data are expressed as means ± SE. Predicted RSA respect to *h *(from *Equation (2) and (3)) *is also plot in the graph. (C) Relationship between thread turns and cell area change (%). The areas of 15 cells were measured in four different microcope fields. Data are expressed as means ± SE. The predicted substrate area change (%) is also plot in the graph. (D) Representative image of phase contrast micrographs (magnification 200×) of HC11 cells attached to silicone membranes. Silicone membranes were progressively subjected to 0% (upper panel), 20% (middle panel) and 30% (lower panel) RSA and the same field is shown in each panel. At each RSA, the arrow points out a loosely attached cell, while the arrowhead shows a well attached one. On the left, contours of cells identified on the right are depicted. Relationship between thread-turns and RSA: 1 turn = 0.54 mm.

We built a theoretical model that let us to predict the strain exerted on flexible membranes (Figure 2). In this geometrical representation the area outlined by the O-ring was considered the "stretchable surface" and it is shown in the diagram as the circle of radius *a *("Initial Position" in Figure 2). In addition, the area defined by the indenter ring is depicted as a circle radius *b*. When the flange (item #4) is pushed down an *h *distance, the membrane adopts an "inverted hat" shape of radius *a*, at the top, and *b*, at the bottom ("Final Position" in Figure 2). This shape was theoretically re-defined as a new circle of radius *c*, whose area could be calculated by *Equation 1*. Then, the relative radial increment of the membrane (*c/a*) could be calculated by *Equation 2 *(Figure 2, grey box).

### Microscopy and Image Analysis

Observations were performed using an inverted microscope (Diavert, Leitz Wetzlar, Germany) equipped with a Canon A520 or a Canon Rebel 350 XT digital camera (Canon USA Inc., USA). The obtained phase contrast video images could be then analyzed by the Image J 1.37v software (NIH, USA).

### RNA preparation, cDNA synthesis and quantitative real-time RT-PCR

Total RNA extraction from HC11 cells was performed using Trizol reagent (Invitrogen, Argentina). For each assay, 1 μg of total RNA was reversed-transcribed as previously described [15]. Real-time PCR data were acquired and analyzed with an Opticon Monitor System (MJ Research, Bio-RAD, Hercules CA, USA). For *c-fos *detection, the primers used were: sense 5'-TCCCTGGATTTGACTGGAGGTCTG-3' and antisense 5'-CGACTGAGGAAGGGTTCG-3'. The thermal cycling conditions were: 95°C for 3 min, followed by 34 cycles of 30 sec at 95°C, 45 sec at 68.7°C for annealing, and 45 sec at 72°C for extension. Levels of *c-fos *expression were normalized by *gapdh *expression that was determined by using the following primers: sense 5'-AAGAAGGTGGTGAAGCAGGCATC-3', antisense 5'-CGAAGGTGGAAGAGTGGGAGTTG-3'. The cycling conditions used were: 95°C for 3 min, followed by 35 cycles of 40 sec at 95°C, 40 sec at 65°C and 40 sec at 72°C. For *lif *and *actin *expression analysis, primers and protocols were previously described [15]. To determine mRNA expression levels, calibration curves were made. Melting curve analysis was performed to confirm a single amplification product. Experiments were always run in triplicate and repeated at least three times.

### Protein analysis

Total or nuclear proteins were extracted from HC11 cells as previously described [15]. Proteins extracts were run in 12.5% SDS-polyacrylamide gels and analyzed by western blot. A set of pre-stained molecular mass standards was run in each gel. Membranes were incubated overnight at 4°C with the following primary antibodies: anti-c-fos (sc-7202), anti-tubulin (sc-9104), anti-pERK (sc-7383), and anti-ERK (sc-154) anti-AKT (sc-1618), anti STAT3 (sc-482), anti-pSTAT3 (sc-8059) from Santa Cruz Biotechnologies (CA, USA) and anti-p-AKT (Cell Signaling Technology, Beverly, MA, USA). After washing, membranes were incubated with horseradish peroxidase-conjugated secondary antibody. Immunoreactive protein bands were detected using the enhanced chemioluminescence system (ECL+Plus System; GE, Buckimghamshire, UK) and the FujiFilm ImageReader LAS-1000. Images were analyzed by densitometry using the Image J 1.34 software (Wayne Rasband, National Institutes of Health, USA. ). For band quantification, the obtained images were converted to grayscale and equal areas encompassing each band were drawn. Then, the integrated density in each rectangle was obtained and the background noise was subtracted for each band. In each case, the obtained value was normalized as indicated in each experiment.

### Enzyme-linked immunosorbent assay (ELISA)

After carrying out stretching protocols, conditioned medium from stretched and control cells was collected and the Leukemia Inhibitory Factor (LIF) content was determined using a mouse LIF-enzyme-linked immunosorbent assay (ELISA) (R&R Systems, Minneapolis, MN, USA) according to the manufacturer's instructions.

### Statistics

Data are expressed as means ± SE. Data were analyzedby one-way ANOVA (following Bartlett's test of homogeneity of variance) followed by Tukey-Kramer as post-hoc test correction for multiple comparisons between means. Statistical comparisons were performed using Statistica (version6.0) software package (StatSoft, Inc., Tulsa, OK, USA). Differences were regarded as significant at p < 0.05. Each experiment was performed independently at least three times.

## Results

The goal of this study was to determine whether applying controlled strain to cultured mammary epithelial cells would induce involution-associated cellular events. The device described herein is capable of delivering up to 30% of two-dimensional homogeneous strain to silicone membranes that were either purchased (Flexcell Inc, NC, USA) or made in the laboratory. The device complete description and assembly process are described in "Methods" and in Figure 1A and 1B. Specifications for the apparatus reproduction are detailed in Figure 1C.

As the flexible membrane remains in the same horizontal plane during strain application, the silicon support and the attached cells can be observed under an inverted microscope before and after stretching. In order to predict the percentage of radial strain applied (RSA) to the elastic substrate, we have developed a theoretical model based on the device geometry (see "Methods"). To test the resultant formula *(Equation 2*, Figure 2A), the distance between pairs of sub-confluent HC11 cells (400–500 μm), were determined before and after application of different strain intensities (*i.e*. different "h" values in *Equation 2*: from 0.285 mm to 6 mm). The homogeneity of the equibiaxial strain was verified by determining the distance between 10 pairs of cells in each orientation (horizontal, vertical and diagonal) in four different microscope fields. Figure 2B shows that the orientation in which cell distances were measured was irrelevant since the increment pattern was almost the same in the three directions. This indicated that the surface of the elastic membrane increased in a homogeneous way throughout flange displacement. Then, to determine whether mechanical strain was efficiently transmitted from the elastic substrate to the attached cells, the area of polygonal (well attached) vs. round (loosely attached) HC11 cells at increasing levels of RSA was analyzed. Only the surface of polygonal attached epithelial cells resulted affected by membrane stretching (Figure 2D, arrowheads), while not significant changes were observed in round cells (Figure 2D, arrows). Then, the area of 15 cells in four different microscope fields, were measured at increasing strain intensities. Figure 2C shows the similarity between the actual and the predicted cell area change (by *Equation 1*) relative to the flange displacement (or screw-top thread turns). These observations indicated there was an efficient strain transmission from the silicone substrate to the mammary epithelial cells. Once this was verified, we carried out the studies described below in confluent HC11 cells grown on the flexible membranes.

**Figure 3:**
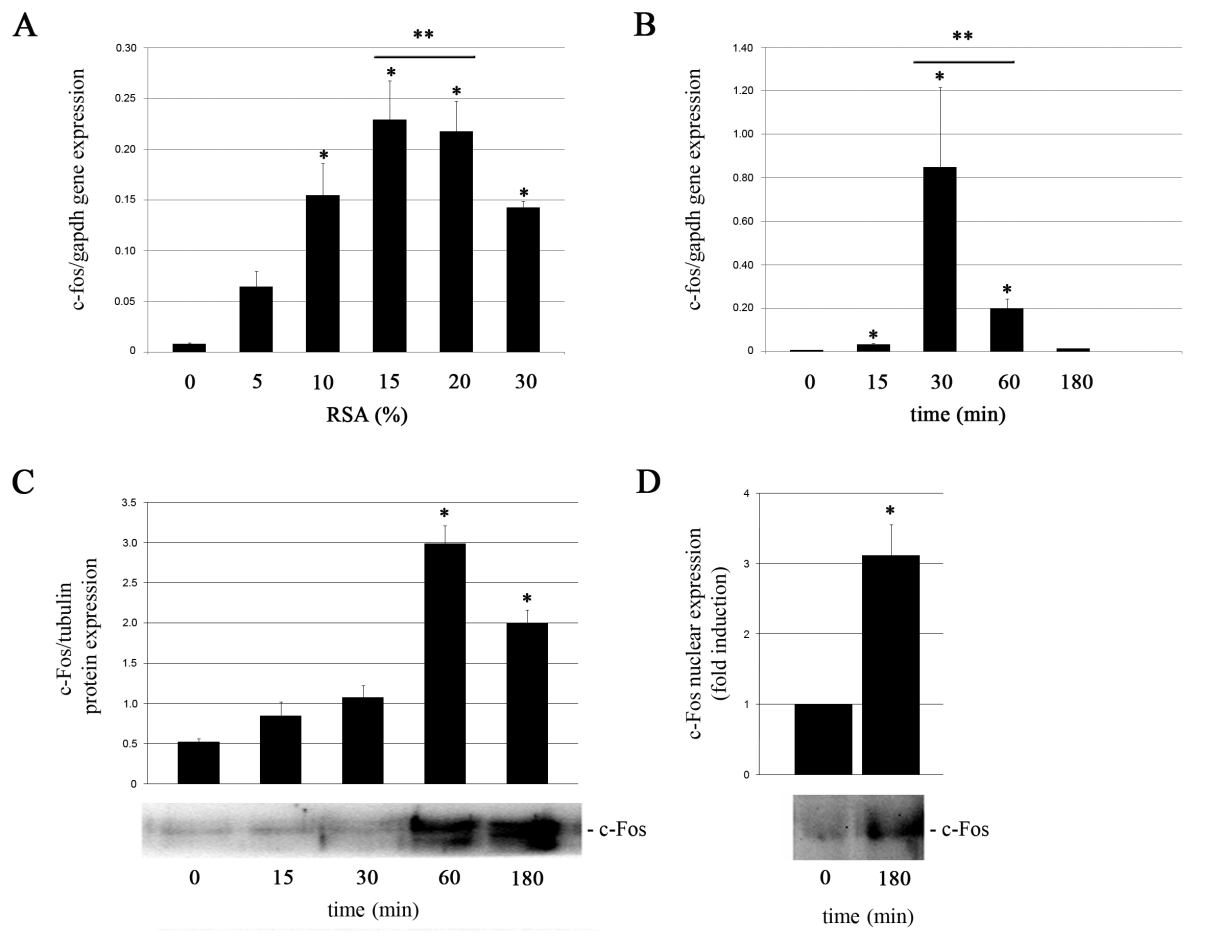
**Effects of mechanical stress on c-Fos expression**. mRNA levels were analyzed by quantitative real time RT-PCR in HC11 cells stretched for 1 h using different strain intensities (A) or subjected to 20% RSA for the indicated time periods (B); *gapdh *expression was used to normalize *c-fos *mRNA levels. c-Fos protein levels were analyzed by western blot in HC11 cells subjected to 20% RSA for the indicated times (C). Nuclear fractions from HC11 mammary epithelial cells were analyzed by western blot to determine c-Fos protein presence in this compartment after 3 h of sustained 20% RSA (D). Analysis was performed by triplicate in at least three independent experiments. Significant differences: (*) p < 0.05 compared to 0% RSA and (**) p < 0.05 compared to 5% RSA.

Since c-Fos protein has been implicated in both, cellular response to mechanical stress and mammary gland involution, we proceeded to analyze its expression and activation levels in our experimental setting. Figure 3A shows that *c-fos *mRNA expression was induced in a dose-dependent manner under rising single-step RSAs (from 0 to 15%) for 1 h. This induction reached a plateau from 15% to 20% RSA and started to decrease at 30% RSA. Then, time course of *c-fos *mRNA expression in HC11 cells subjected to 20% linear strain was analyzed. We found that the highest expression levels were reached after 30 min of sustained strain (Figure 3B). When c-Fos protein expression levels were determined by western blot analysis, a significant increase was observed after 60 min of 20% RSA (Figure 3C). In addition, under these conditions, nuclear localization of the protein was also verified (Figure 3D) indicating that mechanical stress also induced c-Fos nuclear translocation.

**Figure 4:**
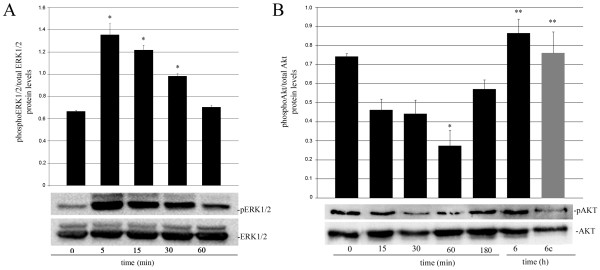
**Effects of mechanical stress on ERK1/2 and AKT phosphorylation**. ERK1/2 (A) and AKT (B) phosphorylation levels were analyzed by western blot in HC11 cells subjected to 20% RSA for the indicated times. Analysis was performed by triplicate in three independent experiments. Data are expressed as means ± SE. Significant differences: (*) p < 0.05 compared with 0 min and (**) p < 0.05 compared with 60 min. Six-hour stretched cells have their own corresponding un-stretched control (grey bar).

It has been reported that the activation of the MAP kinase ERK1/2 is involved in mechanical stress-induced *c-fos *expression and activation in different cell types [16] and its phosphorylation has been observed during the early phase of mammary gland involution *in vivo *[17]. To examine the ability of mechanical strain to induce this MAPK activation in mammary epithelium, HC11 cells were subjected to 20% RSA and ERK1/2 phosphorylation levels were analyzed by western blot. Our results show that mechanical strain was able to induce transient ERK1/2 activation in mammary epithelial cells, achieving the highest phosphorylation level after 5 min of applying such a stimulus (Figure 4A).

In order to explore if mechanical stress could regulate the AKT/PKB surviving signal in mammary epithelial cells, AKT phosphorylation levels in HC11 subjected to mechanical strain were analyzed by western blot. Figure 4B shows that p-AKT levels were reduced after 15 min of 20% sustained strain and were significantly down-regulated at 60 min. Interestingly, basal AKT phosphorylation levels, were fully recovered after 6 h of sustained strain.

**Figure 5:**
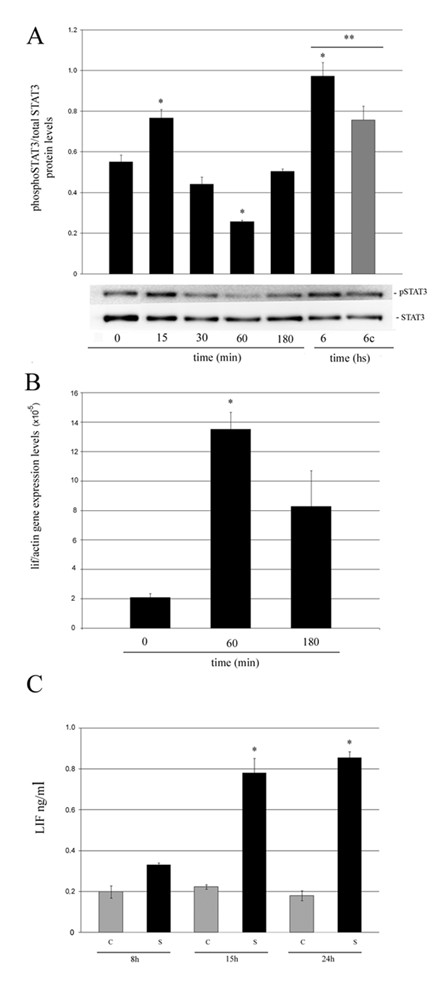
**Effects of mechanical stress on STAT3 phosphorylation and LIF expression**. STAT3 phoshorylation levels were determined by western blot analysis (A) and *lif *mRNA expression levels were analyzed by quantitative real time RT-PCR (B) in HC11 cells subjected to 20% RSA for the indicated times. In (A) and (B), analysis were performed by triplicate in three independent experiments. Data are expressed as means ± SE. Significant differences: (*) p < 0.05 compared with 0 min and (**) p < 0.05 comparing cells stretched for 6 h with its corresponding control (grey bar). LIF expression levels quantified by ELISA in the conditioned medium (CM) of cells subjected (S) or not (C) to 20% RSA for the indicated times. Results are expressed in ng/ml and represent the mean ± S.E. of three independent experiments. Significant differences: (*) p < 0.05 compared to their corresponding control and to CM of cells stretched for 8 h (C).

The activation of the JAK/STAT3 pathway is critical for the initiation of apoptosis and involution in the mammary gland. Therefore, we decided to explore the effects of mechanical strain on STAT3 tyrosine phosphorylation levels by western blot analysis. Figure 5A shows that mechanical stress induced a transient, but significant increase in STAT3 phosphorylation after 15 min of 20% sustained strain. Then, a second STAT3 activation peak was observed after 6 h of applied mechanical strain (Figure 5A). As LIF is the most relevant activator of STAT3 *in vivo *[8,9], we wondered whether this cytokine expression could be induced by mechanical stress in mammary epithelial cells. Therefore, we analyzed the time course of *lif *mRNA induction by real-time PCR. The results show that 60 min of 20% RSA generated a 6 fold up-regulation of *lif *expression in these cells (Figure 5B). To test whether mechanical strain was also able to induce LIF secretion, the presence of this protein in the Conditioned Media (CM) collected from cells subjected or not to 20% RSA for 8 h, 15 h, and 24 h, was analyzed by a LIF-enzyme-linked immunosorbent assay (ELISA). We found that LIF secretion induction was already detected at 8 h and became significant at 15 h and 24 h, compared to 8 h and to their respective not-stretched controls (Figure 5C).

In order to evaluate if mechanical strain was able to induce apoptosis in mammary epithelial cells, caspasa-3 activation was analyzed in HC11 cells subjected to 8 h, 15 h and 24 h of 20% RSA. This analysis was performed by measuring p-nitroanilide levels cleaved from the synthetic substrate Ac-DEVD-pNA. We found that, under these conditions, caspase-3-like activity was not induced in stretched HC11 cells (data not shown).

## Discussion

The mammary gland epithelium undergoes dramatic changes during the successive stages of pregnancy, lactation and involution. In the fully lactating gland, the layer of ductal and alveolar mammary cells form coherent sheets in which cell junctions provide a compact permeability barrier. It has been indicated that mechanical stress imposed by milk secretion on these tightly attached cells after parturition is one of the main factors that determine initiation of the ejection reflex [18]. On the other hand, soon after weaning, when those epithelial junctions are still intact, milk accumulation might initiate involution-associated events.

In an effort to understand mechanical strain contribution to mammary gland involution, we have designed, built and validated a new cell-stretching device. The apparatus presented in this study is inspired in the system previously described by Lee and coworkers [12], but it allows a wider RSA range. Here we show that this device is useful to apply dose-dependent, homogeneous equibiaxial strain to mammary epithelial cells growing on deformable silicone membranes. Further, mechanical strain transmission to the cultured cells was confirmed by cell area changes shown in Figure 2C and 2D. These results show, as it was previously demonstrated in other models [12], that the strain applied to the collagen-coated silicone membranes is very similar to the one exerted to the cells cultured on these membranes. Interestingly, we found relatively larger errors in the percentage of cell area change with RSAs higher than 20% (4 thread turns) (see Figure 2C). Microscopic observations suggested that these relatively larger statistic errors might be due to variations among cells in the ability to adapt to high strain intensities. Excessive cell stretching seemed to cause changes in the attachment of various cells, which could lead to a lower strain transmission from the substrate to these cells (so their area did not increase at the same rate) and/or to an uneven strain distribution within them (causing changes in cell shape).

Different devices that use elastic membranes substrates to support cell adhesion and transmit strain have been previously described [12,19-22]. Some of them, like the one made commercially available by Flexcell International, depend on a computer controlled vacuum unit to exert cyclic or static strain to flexible membranes [23,24]. However, even providing a very precise stimulus control, these devices are complex and often require technical and/or electronic assistance, what leads to a significant cost increment. Therefore, we believed that there was a need for new practical, flexible and affordable devices to easily analyze the biological responses of cultured cells to mechanical stress. In fact, not only our device has been developed as a result of this necessity, there were others very recently introduced, as the one presented last year by Rhana *et al*. [25]. In this apparatus, flange displacement was also used to generate membrane deformation, but the design was made for 6-well plates instead of single 60 mm dishes.

The device described herein shows the following properties: 1) easy real time optical monitoring of cell geometry, function, and deformation upon strain application; 2) a small size that allows long-term stretching protocols to be performed in a cell incubator; 3) easy sterilization by autoclaving and 4) low cost, which permits the construction of multiple units that can be used to test simultaneously different experimental conditions (*e.g*. strain levels, intervals of static stress, pharmacological treatments, etc.).

The intensity of mechanical strain imposed on mammary epithelium by milk accumulation after weaning is not known. However, in most experiments reported herein, a 20% RSA was applied. We have chosen that stretching strength because it did not cause any cell damage and was sufficient to activate various signaling pathways in different cell types [26-30]. Besides, other studies carried out in epithelial cells have been done using 20% RSA [31]. In our laboratory, observations *in vivo *are underway trying to establish the strain range experienced by the alveolar cells upon weaning. We hope this analysis will help us to determine the biological threshold for triggering mammary involution associated events.

Once the device was developed, we first analyzed the effect of mechanical strain on c-Fos expression in the HC11 mammary epithelial cells. This protein interacts with the Jun family members generating AP-1 transcription factors that bind to DNA in specific regions [32]. AP-1 plays a very important role in controlling expression of different genes that have a great impact on cell fate decisions [33-37]. In addition, this factor has been associated with stress-induced apoptosis in several cell types [38]. Marti and colleagues have reported that AP-1 expression and activation is linked to apoptosis induction during the early phase of the mouse mammary gland involution. They observed that *c-fos *and *junB *mRNA species were induced in the mammary epithelium soon after weaning, suggesting that AP-1 may be a nuclear regulator of post-lactational involution [39]. Interestingly, it has also been reported that c-Fos expression is up-regulated by mechanical stress, *in vitro *and *in vivo*, participating in different specific cellular responses in cardiomyocytes [40,41] osteoblasts [42]and pulmonary epithelial cells [43]. Our results clearly indicate that mechanical strain is capable of inducing c-Fos transcription and nuclear translocation in mammary epithelial cells, suggesting that they might be very early events during the involution process.

Three Mitogen-activated protein kinase (MAPK) families have been well characterized: the extracellular-regulated protein kinase (ERK1/2), the c-Jun NH2-terminal protein kinase (JNK), and the p38 (the last two are also known as stress-activated protein kinases or SAPKs). Upon activation through tyrosine and threonine phosphorylation, these proteins translocate to the nucleus and phosphorylate transcription factors, such as AP-1 family members and the serum response factor (SRF) [44]. MAPK activation, have been implicated in mechanically induced signalling in various cell types. For example, in smooth muscle cells, ERK1/2 has been reported to be involved in mechanical stress-induced c-Fos expression [16]. Interestingly, ERK1/2 activation was also detected during the early phase of mammary gland involution *in vivo *[17]. Our results indicate that ERK1/2 phosphorylation is rapidly increased in mammary epithelial cells subjected to the same mechanical strain that generated c-Fos induction. Therefore, it is possible that ERK1/2 activation induced by mechanical strain may be modulating c-Fos expression induction and activation as previously observed in other models [16,45,46].

Not only ERK1/2, but also STAT3 might be implicated in c-Fos expression induction and activation in epithelial cells [47]. In the case of c-*fos *gene transcription, it has been determined that both STAT3 and the ERK-mediated pathway co-operate in its induction. In fact, Kunisada *et al*. [48] showed that dominant-negative STAT3 or a MEK inhibitor, PD98059, inhibit LIF-induced c-*fos *mRNA expression in cardiac myocites. Therefore, the same signaling pathways might be interacting in mechanically stressed mammary epithelial cells.

One of the most critical molecular changes associated with apoptosis induction during mammary gland involution is STAT3 activation via the Janus kinase (JAK) pathway in response to cytokines and growth factors [17,49]. It has been demonstrated that shortly after weaning, LIF expression is induced in the mammary epithelium [8,9] and it has been reported that this cytokine is the main responsible for STAT3 activation in mammary epithelial cells [8,9,15]. Interestingly, it has been shown that LIF expression is induced by hemodynamic overload in the adult mammalian heart [50] and our results show that cell stretching induced STAT3 phosphorylation after 15 min of sustained strain. However, LIF secreted by the stretched HC11 cells was only detected in the conditioned medium (CM) after several hours of sustained strain (see Figure 5C). Therefore, we believe this STAT3 early activation would not be due to an endocrine/paracrine LIF action. Alternatively, it is possible that SRC kinase might be involved in p-STAT3 induction in the HC11 cells as it has been previously found in mechanically induced pulmonary epithelial cells [51] and smooth muscle cells [52].

Levels of p-STAT3 significantly decreased after 1 h and were recovered after 6 h (Figure 5A). We believe that the second wave of STAT3 activation might be due to LIF secreted by the stretched cells (see Figure 5C). In fact, preliminary data from our laboratory indicates that CM collected from stretched cells (6–24 h of 20% sustained strain) induced STAT3 phosphorylation in non-stretched HC11 cells. Noteworthy, this effect was inhibited when CMs were pre-incubated with a LIF blocking antibody.

Several studies regarding the impact of mechanical stimuli on protein kinase B/AKT (PKB/AKT) activation have been described in endothelial cells [53], in vascular smooth muscle [54] and keratinocytes [55]. In this study, we observed that mechanical strain triggered p-AKT transient down-regulation in mammary epithelial cells (see Figure 4B). In the mammary gland, the relevance of shutting down the PI(3)K/AKT pathway after weaning has been demonstrated when activated-AKT transgenic mice showed significant delay in the involution process [56,57]. In addition, it has been reported that expression of the PI(3)K negative regulatory subunits (p55alpha and p50alpha), which inhibited AKT phosphorylation, were induced by STAT3 during mammary regression [58]. These studies indicate that AKT activation may provide a critical cell survival signal that has to be turned-down during mammary involution. Therefore, we believe that stretching induced p-AKT down-regulation might sensitize epithelial cells to undergo apoptosis. However, more experiments need to be done to determine whether or not STAT3 activation mediates this effect in our model.

In spite of p-AKT down-regulation, we have not detected apoptosis induction (analyzed by caspase-3 activation) in the stretched HC11 cells. This observation has different possible explanations. First, it has been reported that in smooth muscle cells, mechanical stress induced apoptosis is p53-dependent [59]. Therefore, the lack of apoptosis induction in the stretched HC11 cells might be solely due to the lack of wild type p53 expression in this cell line [60]. Second, it has been reported that *in vivo *active (cleaved) caspase-3 was observed only in the shed cells at 12 h and 24 h involution and not in the alveolar wall until 72 h [61]. Therefore, during mammary involution, although the apoptosis program is initiated before cells detach, the final events would not occur until these cells are removed from the epithelial layer. In this scenario, it is conceivable that an early involution-associated event as mechanical stress may prepare the epithelial cells to die, but would not be enough to trigger the whole apoptotic program.

It is important to point out that the experiments showed herein were performed in confluent, but not differentiated, HC11 cells. We observed that grown on silicon as a substrate, these cells show similar features to competent HC11 cells grown on plastic. We observed high expression of STAT5A and low levels of p-STAT5 and β-casein compared to cells treated with lactogenic hormones. Expression levels of these proteins did not significantly change upon stretching (data not shown). However, we do not know whether mechanical stress would be able to block the action of lactogenic hormones and/or would be able to trigger cell death in fully differentiated cells. More experiments are being performed to answer these questions.

We have previously reported that tumor cell secreted LIF was able to decrease HC11 cell viability [15]. Here, we show that cells stretched for up to 24 h did not undergo apoptosis, but were able to secrete up to 0.8 ng/ml of LIF (Figure 5C). New experiments carried out in our laboratory have shown that LIF secreted by mechanically stressed cells was able to induce STAT3 phosphorylation in non-stretched cell (data not shown). Therefore, luminal cells bearing mechanical stress might not be the first to die, but could initiate a domino effect that may lead to massive apoptosis in the mammary epithelium.

## Conclusion

The results showed herein provide, for the first time, experimental evidence that mechanical strain applied to mammary epithelial cells induces molecular events involved in the initiation of post-lactational involution. A big advance was made when it was determined that this biological process was not only regulated by circulating hormones, but also -and primordially- by tissue local factors. Similarly, determining that mechanical forces play a relevant role in the initiation of such a complex process might reveal significant mechanisms underlying cell fate decision in the mammary gland. However, studying this scenario requires new experimental approaches involving the development and/or adaptation of methods and apparatus. Therefore, here we have made a special effort to carefully describe the device and the geometrical model we built to such a purpose. Using them we were able to demonstrate that mechanical stress can trigger intracellular pathways that facilitate epithelial apoptosis and secretion of specific cytokines that may induce death in neighboring non-stretched cells.

## Authors' contributions

All authors helped in the design of the cell stretching apparatus. In addition, each of them made the following specific contributions: AQ designed the protocol to prepare the silicone membranes, set up the cell culture conditions, performed the real rime RT-PCR and western blot techniques, made the statistical analysis and graphics of the obtained results and wrote the manuscript draft; MS and JP designed the geometrical model to predict stretching intensity, designed and performed visual analysis of cells and substrates and helped to improve the quality of the lab-made silicone membranes; NR performed the ELISA for LIF and ECK proposed the initial idea of testing the relevance of mechanical stress in inducing mammary involution-associated events; designed and coordinated the biological experiments and wrote the final manuscript. All authors read and approved the last submitted version.
